# Dual-Mode Wheat Germ Agglutinin Labeling – A Versatile Cell Segmentation Strategy for High-Resolution LA-ICP-TOFMS Bioimaging

**DOI:** 10.1021/acs.analchem.5c04060

**Published:** 2025-09-18

**Authors:** Claude Molitor, Martin Schaier, David Loibnegger, Gabriel Braun, Michael Gutmann, Walter Berger, Gunda Koellensperger

**Affiliations:** † Institute of Analytical Chemistry, Faculty of Chemistry, 27258 University of Vienna, 1090 Vienna, Austria; ‡ Institute of Inorganic Chemistry, Faculty of Chemistry, University of Vienna, 1090 Vienna, Austria; § Vienna Doctoral School in Chemistry (DoSChem), University of Vienna, 1090 Vienna, Austria; ∥ Center for Cancer Research and Comprehensive Cancer Center, 27271 Medical University of Vienna, 1090 Vienna, Austria

## Abstract

Single-cell analysis by laser ablation inductively coupled plasma time-of-flight mass spectrometry (LA-ICP-TOFMS) enables high-resolution mapping of elemental distributions and cellular phenotypes. Segmentation of individual cells necessitates labeling of both nuclei and membranes, the latter often requiring extensive tissue-specific optimization. In this study, we present a broadly applicable segmentation protocol based on wheat germ agglutinin (WGA), a lectin that binds to *N*-acetylglucosamine and sialic acid residues ubiquitously expressed on the cell membrane. By combining fluorescently labeled WGA with a metal-tagged anti-WGA antibody, we introduce a dual-labeling strategy compatible with both fluorescence microscopy and LA-ICP-TOFMS, enabling cross-validation of membrane labeling and enhancing segmentation accuracy. With recent advancements in laser ablation technology, such as higher repetition rates and submicrometer spot sizes, high-resolution imaging across large sample areas has become increasingly feasible. The robust, high-contrast membrane labeling achieved with our method facilitates precise cell segmentation at these resolutions and enhances the quality of the downstream single-cell data analysis. Beyond that, our approach reduces staining costs, streamlines workflows, and provides a scalable alternative to existing membrane-labeling strategies.

## Graphical abstract

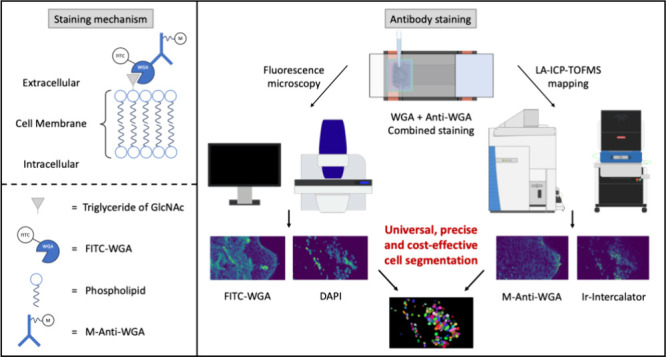

## Introduction

Single-cell analysis has transformed our understanding of biological complexity by enabling detailed investigations into cellular heterogeneity and the effects of environmental factors, diseases, and therapeutics on individual cells within tissues. Laser ablation inductively coupled plasma time-of-flight mass spectrometry (LA-ICP-TOFMS) has emerged as a powerful platform for spatial single-cell analysis, allowing for unprecedented exploration of tissue-level and cell-level heterogeneity. In LA-ICP-MS, a pulsed laser beam ablates material from the sample, which is transported by inert gas to the ionization source, ionized, and subsequently analyzed by mass spectrometry.^1^ Elemental maps are obtained by scanning across the sample. The advent of bioimaging by elemental mass spectrometry can be traced back to 1994.^2^

Since then, substantial advancements in instrumentation and methodology paved the way to spatial multiplexed analysis at single-cell level. Key instrumental milestones include (1) the introduction of time-of-flight mass spectrometry with spectral acquisition rates >20 kHz allowing for multiplexing and (2) the development of low-dispersion laser ablation cells, key for high spatial resolution. The present configuration of low-dispersion laser ablation cells is capable of generating and efficiently transporting material plumes at millisecond scale, following the process of ablation.^2–4^ The utilization of short pulse response times has been demonstrated to enhance sensitivity and support pulse-resolved mapping, a prerequisite for spatial resolutions down to <1 μm. Finally, minimizing pulse response times allows for high laser repetition rates, thereby ensuring high throughput. Today, pulse responses of ≤1 ms can be achieved.^3,5^ The latest generation of deep ultraviolet lasers has been demonstrated to be capable of operating at a rate of up to 1000 Hz while maintaining a beam profile of 1 μm.^6,7^ Furthermore, through oversampling, even smaller pixel sizes can be achieved. As a drawback, the established routine of pulse responses optimization relies on NIST glass reference material, showing markedly different ablation behavior than biological samples. Consequently, the short pulse responses <1 ms observed for glass, are not for granted when applying the same experimental conditions on biological materials. Achieving both high acquisition speeds and single-cell resolution remains challenging. At higher throughputs, additional factors such as element-specific pulse responses and the demand for rapid multielement data processing increasingly influence the accuracy of imaging. Thus, although instrumental developments enable high-throughput imaging with submicrometer detail, most state-of-the-art bioimaging studies still report pulse response times of 1–5 ms, repetition rates below 500 Hz, and spatial resolutions around 1 μm. Recently, Voloaca et al. reported multiplexed bioimaging of malignant mesothelioma tissue with pixel acquisition rates of 250 Hz and spatial resolutions of 2 μm.^8^ Bost et al. performed bioimaging of melanoma tissue at 800 Hz with 1 μm resolution.^9^ The smallest reported spot size to date is 0.6 μm by Van Malderen et al. with elemental maps acquired at 100 Hz, a shot count of 2 resulting in a pixel acquisition rate of 50 pixel/s, without immunostaining and thus no single-cell analysis.^4^

Introducing the toolbox of metal-based immunohistochemistry (IHC) was the essential methodological step for single-cell analysis by ICP-MS.^10^ In fact, label-free LA-ICP-MS bioimaging precludes single-cell analysis within the tissue. Endogenous elements, such as sulfur and phosphorus, do not provide sufficient contrast to identify cellular boundaries or nuclei. State-of-the-art imaging mass cytometry (IMC) covers comprehensive panels of up to 60 (routinely 40) metal-labeled antibodies probing target biomolecules and cells, thus allowing for in-depth phenotypic characterization of tissues. The protocols involve membrane and nucleus staining, as a prerequisite to extract information on cell type, function, and state at single-cell level. The process of assigning pixels to cells is known as cell segmentation. Accurate cell segmentation is the prerequisite for downstream image analysis. While nucleus staining is established by a DNA-intercalating metal species (Ir), membrane labeling relies on metal-labeled antibodies and requires tailored IHC across different tissues. To date, identifying the precise cell boundaries for segmentation poses a challenge to many IMC studies. Platinum-based segmentation kits were introduced, emphasizing the need for a universal membrane labeling strategy. Although commercially available segmentation kits simplify the segmentation process, they still require manual optimization while failing to achieve full coverage of cell types and are proprietary and accompanied by high costs. As a result, the platinum label is fixed and cannot be easily exchanged for other metal isotopes.

In this work, we propose a novel membrane metal-labeling strategy based on wheat germ agglutinin (WGA). WGA is a lectin molecule that binds to *N*-acetylglucosamine and sialic acid residues on cell membranes. This lectin chemistry enables universal membrane labeling across diverse cell types, eliminating the need for tissue-specific markers. WGA labeling is well established in Immunofluorescence (IF) and has proven compatible with a wide range of sample types, including FFPE tissue sections, cryosections, and 2D cell cultures. Additionally, this approach is cost-effective and robust across different sample preparation protocols. Unlike protein epitopes, WGA-binding glycans are unaffected by enzymatic treatments, such as trypsinization, ensuring consistent labeling in cell cultures. The use of WGA membrane labeling in IMC is still new. So far, only one study reported on metal-labeled WGA. Free thiol groups were introduced on WGA via Traut’s reagent, allowing for direct attachment of metal polymer tags. The method was proposed for barcoding in single-cell ICP-MS.^11^ In contrast, our study introduces a dual-labeling approach for WGA that enables the seamless integration of IF and mass spectrometry imaging. Instead of directly attaching metal tags to WGA, fluorescently labeled WGA is incubated with a metal-tagged anti-WGA antibody. This dual-labeling strategy offers a powerful tool for validating IMC data through IF. We showcase it through multimodal imaging of skin tissue and 2D cell cultures, achieving highly sensitive membrane labeling and producing high-quality single-cell elemental maps at unprecedented submicrometer resolution. These improved images bring IMC closer to the quality of IF, setting new standards for the technique.

## Experimental Section

The Supporting Information provides detailed descriptions of chemicals and materials, human skin sample preparation, HCT116 cell cultivation and treatment, LA-ICP-TOFMS instrumentation, data acquisition, and processing.

### Tissue Staining Protocol

Tissue samples were deparaffinized in fresh xylene for 20 min and rehydrated through a graded ethanol series (100% to 70%). After thoroughly rinsing with ultrapure water, heat-induced antigen retrieval was performed in Tris-EDTA buffer (pH 9), at 96 °C for 30 min. Sections were then cooled to RT and washed with a TBS buffer containing 0.05% Tween to permeabilize the tissue.

Nonspecific antibody binding was reduced by sequential incubation with SuperBlock blocking buffer for 30 min at room temperature (RT), followed by Human BD Fc Block for 10 min. Antibody staining was performed overnight at 4 °C in a hydration chamber.

The next day, FITC-labeled WGA (F-WGA) and metal-labeled anti-WGA (M-Anti-WGA) were mixed at equimolar concentrations and preincubated for 2 h at RT in TBS/0.05% Tween, diluted 1:10. After preincubation, TBS/0.05% Tween was added to the mixture to achieve the desired dilution (1:25 to 1:200, depending on the application such as high- or low-resolution imaging). For a recommended dilution of 1:100 for six skin samples, 0.5 μL of F-WGA, 1.5 μL of M-Anti-WGA, and 23 μL of TBS/0.05% Tween have been preincubated for 2 h. For membrane staining, 125 μL of TBS/0.05% Tween was added to the preincubated mixture to achieve the desired 1:100 M-Anti-WGA dilution. After the samples were incubated for 30 min with the mixture, the slides were washed with TBS/0.05% Tween, then incubated with an iridium-based DNA intercalator (1:100) and DAPI staining solution (1:3), both diluted in TBS/0.05% Tween, for 5 min at RT in a hydration chamber. To prevent photobleaching, coplin jars were covered during washing steps. The slides were washed twice in TBS/0.05% Tween, and sections were rinsed thoroughly with ultrapure water to prevent TBS crystallization during the drying process. Stained samples were subsequently analyzed by immunofluorescence (IF) and LA-ICP-TOFMS.

### Cytospin Staining Protocol

In alignment with the tissue staining protocol, the F-WGA/M-Anti-WGA mixture was prepared by preincubating equimolar concentrations in TBS for 2 h at RT. For approximately 1.5 × 10^6^ cells, 2.5 μL of F-WGA and 7.5 μL of M-Anti-WGA were suspended in 30 μL of TBS. Following preincubation, the F-WGA/M-Anti-WGA mixture was added to the cell suspension (120 μL), yielding a final volume of 150 μL. The suspension was incubated for 30 min at RT with occasional vortexing to ensure uniform distribution. After incubation, cells were centrifuged at 1400 rpm, and washed twice with TBS. Cells were deposited onto Superfrost microscope slides (Thermo Scientific) using a Cytospin 4 cytocentrifuge (Thermo Scientific) at 400 rpm for 5 min. The cytospins were air-dried for 20 min before fixation. Fixation was performed by applying 80 μL of 4% paraformaldehyde (PFA) to each cytospin for 20 min at RT. Subsequently, samples were rinsed with ultrapure water. For nuclear staining, the iridium-based DNA intercalator was diluted 1:100 directly into DAPI staining solution and applied to the slides for 5 min at RT in a hydration chamber. Samples were then rinsed with ultrapure water and air-dried. Prepared cytospins were analyzed by IF and LA-ICP-TOFMS.

### Instrumentation

LA-ICP-TOFMS measurements were conducted using an Iridia 193 nm laser ablation system (Teledyne Photon Machines) coupled with an icpTOF 2R instrument (TOFWERK). IF imaging was performed on a Zeiss Axioscope 7 microscope equipped with an Axiocam 305 camera and a Colibri 7 LED illumination system.

## Results and Discussion

### Optimization of WGA Staining

Accurate cell segmentation is critical for extracting reliable cellular data from tissue sections and cell cultures. Improving resolution down to a subcellular level necessitates the intense labeling of highly abundant cellular features in order to maintain detectable signal intensities. This is especially important since smaller spot sizes and increased oversampling result in smaller amounts of ablated sample per shot, demanding higher signal intensity for effective detection. *N*-acetylglucosamine (GlcNAc), a highly abundant glycan on cell membranes, can be targeted by the lectin (WGA). WGA is well-established in immunofluorescence (IF) to define cell boundaries due to its broad activity across cell types. However, the direct labeling of WGA with metal tags presents significant challenges. In particular, disulfide bond reduction, as employed in IMC protocols, compromises WGA’s glycan-binding affinity. We developed a dual-labeling strategy that combines well established fluorescently labeled WGA (FITC or Rhodamine; denoted as F-WGA; in this work, only FITC-WGA data are presented) with a metal-tagged anti-WGA antibody (denoted as M-Anti-WGA) targeting the antigen WGA. This method leverages commercially available metal antibody labeling kits, ensuring high labeling efficiency while preserving the binding specificity of native WGA. Using a dual-labeling approach provides the additional advantage of direct comparison of independently acquired multimodal images supporting validation and expanding analytical capabilities.

The staining procedure was thoroughly optimized, scrutinizing different protocols like separate staining of F-WGA/M-Anti-WGA, simultaneous co-staining, and a combined preincubation-based staining approach. The preincubation protocol produced the best results, reducing the staining time to 30 min at room temperature while maintaining high sensitivity. This also facilitated milder staining conditions compared with conventional antibody-based membrane labeling, which typically requires prolonged incubation and antigen retrieval. Since WGA, being a lectin, targets glycans rather than protein epitopes, antigen retrieval is unnecessary. Furthermore, because WGA itself serves as the antigen for the Anti-WGA antibody, the WGA/Anti-WGA complex can perform during preincubation, thus further shortening the tissue staining time. Details on the optimized staining are described in the Experimental Section.

To reduce reagent consumption while ensuring robust signal output, different staining times and concentrations of F-WGA and M-Anti-WGA were tested. Figure 1 presents these conditions evaluated on human skin tissue sections. Our results show that a 1:100 dilution with a 30 min incubation (Figure 1A) provides optimal staining. When reducing the incubation time to 5 min (Figure 1B), signal intensity is still usable but noticeably reduced. At dilutions as low as 1:200 (Figure 1C), the signal intensity diminishes, yet sufficient cellular information can still be extracted. For high-resolution imaging with submicrometer pixel sizes, F-WGA/M-Anti-WGA concentrations can be increased by diluting the stock solution up to 1:25 to increase sensitivity to compensate the smaller ablated volumes (Figures 1D and 1E).

**Figure 1:**
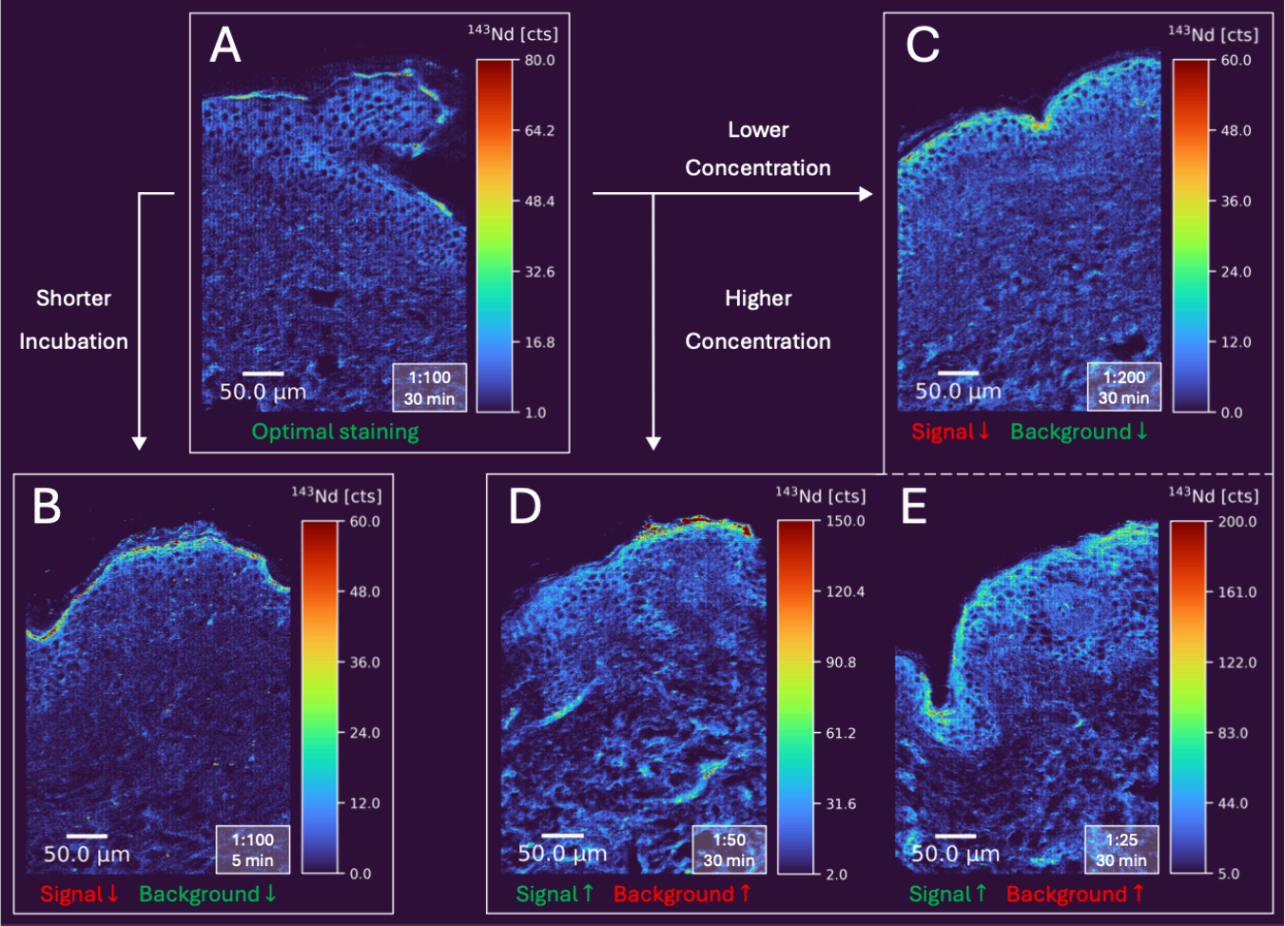
Optimization of the dual-labeling approach for membrane staining. Maps were acquired with LA-ICP-TOFMS at 1 μm resolution (2 μm spot size, 2× overlap in both directions) with a repetition rate of 500 Hz. Green text indicates positive effects, red text negative effects. (A) shows F-WGA/M-Anti-WGA at 1:100 dilution with a 30 min incubation, recommended for staining. (B) uses the same dilution but with a shortened 5 min incubation. Panels (C), (D) and (E) display different F-WGA/M-Anti-WGA dilutions from 1:200 and 1:25, all with 30 min incubations.

### Precise Cell Boundary Delineation in Skin Using Dual-Marker Approach

Using the optimized dual-labeling protocol F-WGA/M-Anti-WGA, skin tissue sections were analyzed by integrating IF and LA-ICP-TOFMS. Previous IMC studies have targeted the cell surface receptor CD44, a hyaluronate receptor for membrane labeling in human skin.^12^ However, comparison of WGA and CD44 images from the same skin section, obtained by LA-ICP-TOFMS (Figure S1), revealed that WGA-based labeling covers a broader range of cell types than CD44. Given the variable expression of CD44 across different cell populations, membrane labeling is inherently heterogeneous. In contrast, WGA binds to glycan structures ubiquitously present on cell surfaces, enabling more uniform, cell-type independent membrane staining (Figure 2S).^13–15^ This broader applicability makes WGA well-suited for imaging complex and heterogeneous tissue environments, supporting comprehensive single-cell analysis by IMC.

The power of the dual-labeling strategy is showcased in Figure 2, presenting IMC and IF of the same skin tissue section. Using instrumental nominal settings, we claim pulse-resolved imaging at pixel sizes below 500 nm, an unprecedented spatial resolution for LA-ICP-TOFMS bioimaging, supported by direct comparison of the two modalities. A routinely established 1 μm circular laser spot together with laser ablation stages with nanometer precision produced effective pixel sizes down to 250 nm (up to 4× overlap in both the *x*- and *y*-directions; controlled via dosage and interspacing). Optimizing the laser fluence, full ablation of the tissue thin section was obtained (5 μm thickness) for every spot, allowing for image reconstruction without data deconvolution. Achieving this submicrometer resolution required a highly sensitive membrane label, as reduced ablation volumes inherently lower signal intensity and raise detection limits. Both labeling modalities, F-WGA and M-Anti-WGA, provided robust and complementary membrane staining. The signal of WGA and anti-WGA, as well as the nuclear markers iridium and DAPI, showed strong spatial correlation, effectively delineating cell boundaries and nuclei. This complementary signal distribution improves the segmentation quality and enables more accurate identification of cellular outlines. Submicrometer-resolution mass spectrometry imaging supports multimodal analysis in an unprecedented manner, facilitating orthogonal cross-validation by IF and enabling robust coregistration for advanced spatial omics workflows.

**Figure 2:**
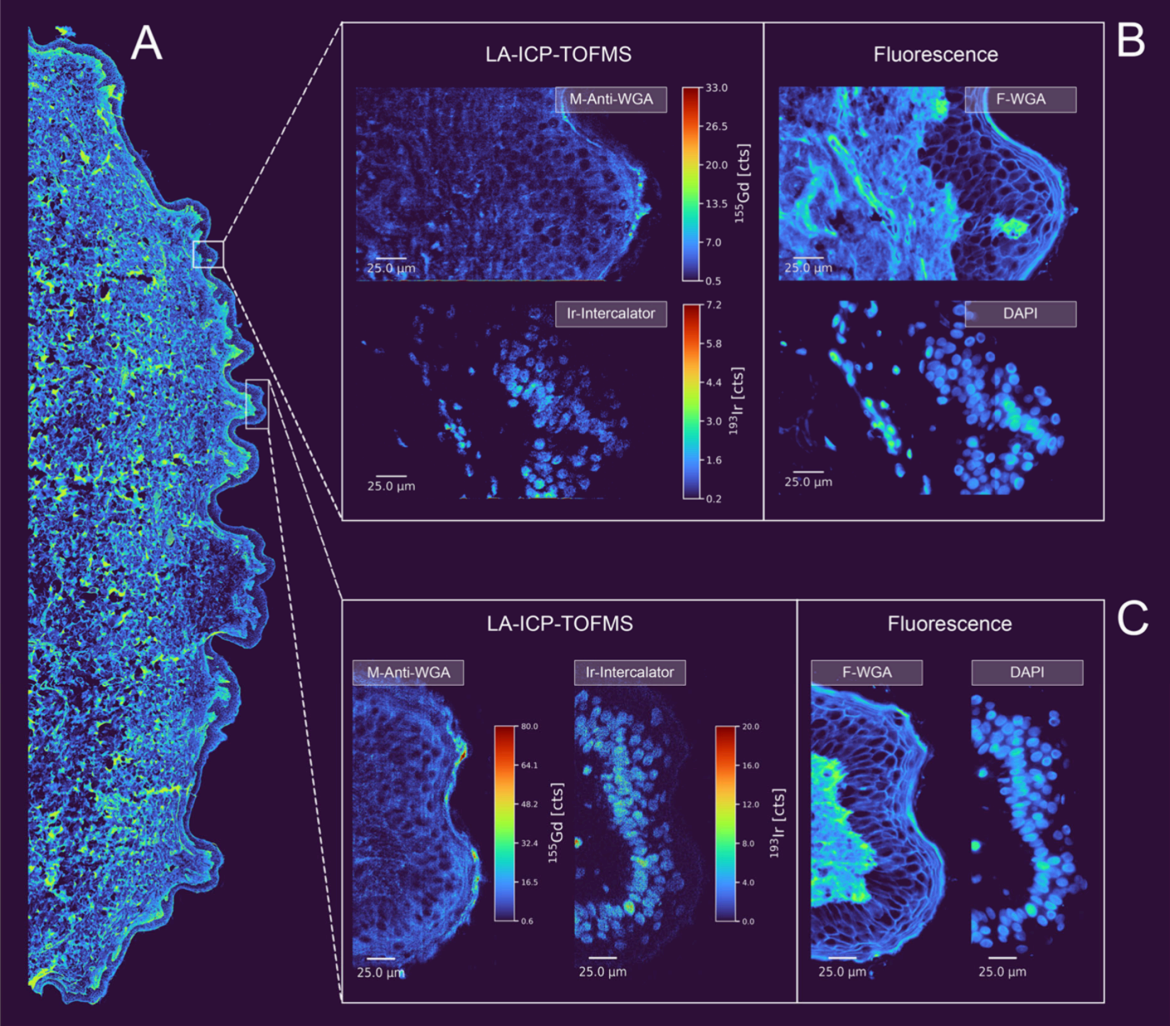
Comparison of IF and LA-ICP-TOFMS images after membrane staining using F-WGA/M-Anti-WGA dual-labeling at 1:25 dilution with 30 min incubation to improve signal for subcellular imaging. Panel (A) shows a large skin tissue area visualized by IF with the membrane marker F-WGA. Panels (B) and (C) show LA-ICP-TOFMS images of M-Anti-WGA and Ir-Intercalator for different epidermal regions, acquired with pixel sizes of 250 nm (panel (B)) and 333 nm (panel (C)) (1 μm spot size with 3× and 4× overlap, respectively, in both directions) at a repetition rate of 250 Hz, alongside corresponding IF images of F-WGA and DAPI. A Gaussian blur filter (σ = 0.6) has been applied to the LA-ICP-TOFMS images.

### Mapping Metal Uptake in Subcellular Compartments of Cytospin Samples

Labeling cytospins presents distinct challenges compared with FFPE sections. A key difference is that cells on the cytospins remain intact and unsectioned, preserving the integrity of the plasma membrane. Consequently, membrane markers label the entire exposed surface of the cell, unlike in FFPE tissue sections, where staining is often restricted to the outermost cell boundaries. This difference results in a more homogeneous staining pattern in cytospins, in contrast to the typical “coffee-stain” appearance in FFPE tissue, which aids in distinguishing tissue morphology.

Figure 3 presents cytospin samples derived from 2D HaCaT cell cultures, aneuploid immortal keratinocyte cells from adult human skin, and HCT116 colon cancer cell cultures. HCT116 cells were treated with the anticancer drug oxaliplatin (OxPt). Initially, the WGA staining protocol optimized for FFPE tissue sections was applied. However, this approach proved suboptimal for cytospins with high cell density, where adjacent cells could not be reliably resolved due to insufficient contrast from the WGA-based membrane staining (Figure 3A, left). To overcome this limitation, a tailored labeling protocol was developed that involves WGA staining of cells in a suspension prior to cytospin preparation. Briefly, adherent cells were trypsinized within the bottom flask, washed and then mixed with a preincubated F-WGA/M-Anti-WGA solution (see details in the Experimental Section). This labeling strategy in a cell suspension enabled comprehensive membrane staining of the entire cell membrane, in contrast to fixed cytospin samples, where only the upper surface is accessible to staining reagents. This approach significantly improved membrane signal intensity, particularly in densely packed regions (Figure 3A, right) facilitating reliable single-cell segmentation in high-density cytospin samples. Unlike antibody-based methods, WGA targets glycans that remain intact after trypsinization, whereas protein epitopes may be cleaved, providing an important advantage for membrane staining.

**Figure 3:**
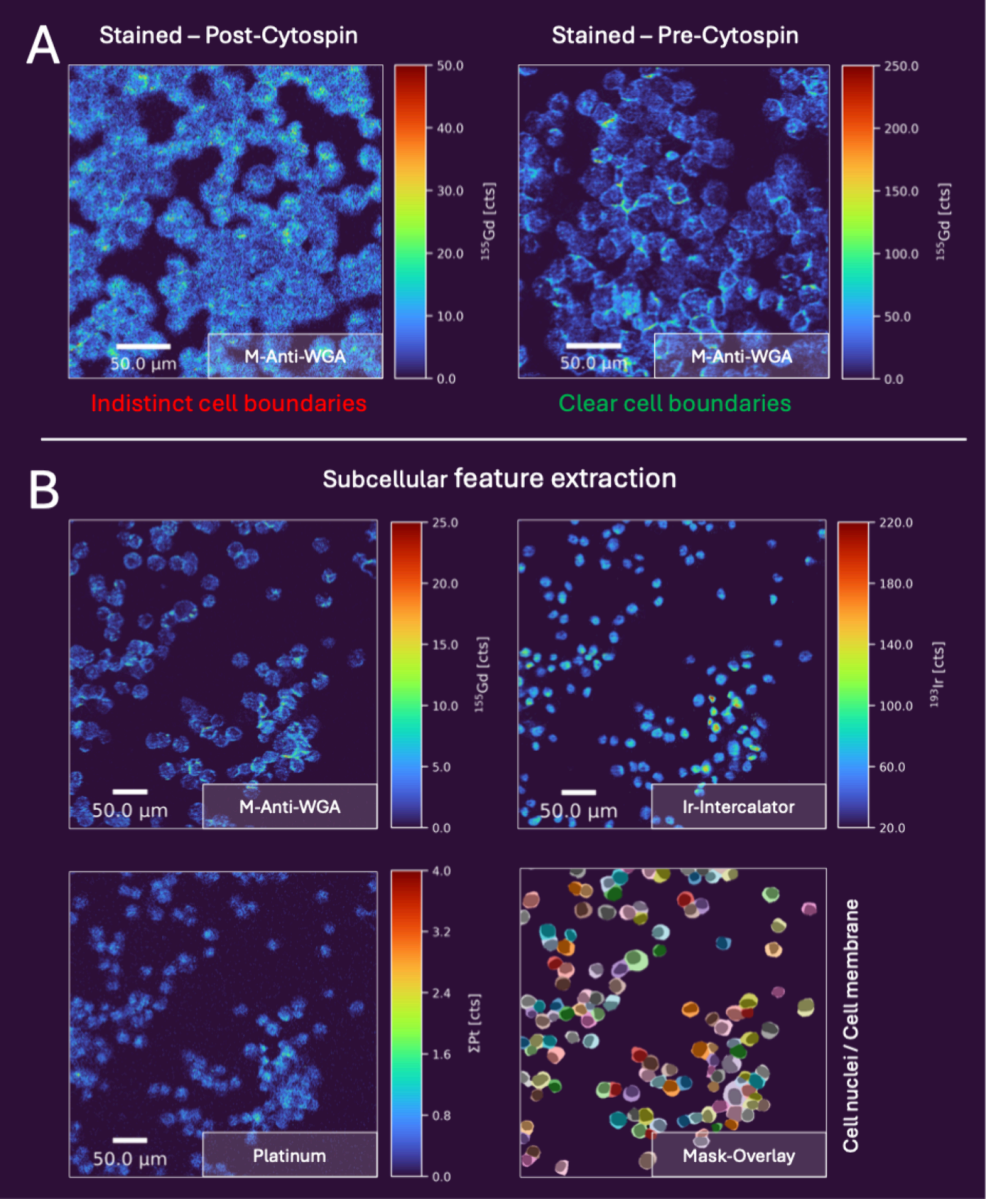
Comparison of F-WGA/M-Anti-WGA membrane labeling strategies for cytospins and their application to subcellular platinum mapping in HCT116 cells. (A) Cell membranes labeled with M-Anti-WGA after cytospin preparation (left, HaCaT) versus precytospin in suspension (right, HCT116). Maps were acquired with LA-ICP-TOFMS at 1 μm resolution (2 μm spot size, 2× overlap in both directions) with a repetition rate of 250 Hz. (B) Maps of cell membrane (M-Anti-WGA), nuclei (Ir-intercalator), and platinum (sum of ^194^Pt, ^195^Pt, ^196^Pt) in HCT116 cytospins treated with 25 μM oxaliplatin for 24 h. Segmentation masks illustrate subcellular compartments (membrane and nuclei overlap). All maps were acquired with LA-ICP-TOFMS at 500 nm resolution (1 μm spot size, 2× overlap in both directions) with a repetition rate of 250 Hz. A Gaussian blur (σ = 0.6) was applied to the platinum image to enhance the image clarity.

Finally, the validity of this approach was showcased by analyzing the metal uptake in subcellular compartments of HCT116 cells following OxPt exposure. Figure 3B presents LA-ICP-TOFMS maps of cell membranes, nuclei, and platinum (Pt) at a spatial resolution of 500 nm. The optimized WGA labeling enabled clear cell segmentation and subcellular feature extraction. Although the relatively low Pt levels in individual cells limited the resolution, 500 nm was sufficient to distinguish the nuclear and cytoplasmic compartments within HCT116 cells. Pt was notably enriched in the nuclear region, consistent with OxPt’s DNA-targeting mechanism.^16,17^

A detailed view is provided in the closeup in Figure S3.

## Conclusions

The F-WGA/M-Anti-WGA dual-labeling strategy provides efficient and robust membrane staining, significantly enhancing cell segmentation, a key step for phenotyping heterogeneous cell populations. By simplifying the workflow and reducing reagent costs, this approach overcomes key challenges in single-cell analysis. Leveraging recent developments in LA-ICP-TOFMS instrumentation, we found the boosted sensitivity in membrane labeling allowed for imaging at subcellular resolution. Finally, the dual-modality of the labeling methods supports the seamless integration of high-resolution LA-ICP-TOF-MS and IF, allowing for cross-validation and more comprehensive data from individual cells through the complementary strengths of both imaging modalities. The versatility and effectiveness of this novel WGA dual-labeling approach were demonstrated across diverse applications including tissue specimen and cell culture models. Finally, the labeling approach introduced here is not restricted to the combination of IF and LA-ICP-TOFMS, but supports alternative high-resolution elemental imaging methods as well.

## Supplementary Material



## Data Availability

The data supporting the findings of this study are provided in the Supporting Information accompanying this article. Upon reasonable request, raw images of the data will be made available. For further inquiries, please contact Gunda Koellensperger at gunda.koellensperger@univie.ac.at.

## Notes

Comparison of WGA and CD44 staining; close-up of WGA and CD44 for regions of interest; detailed view of cellular platinum uptake; instrumental parameters (PDF)
