# Supplementary material for: Dual-Mode Wheat Germ Agglutinin Labeling – A Versatile Cell Segmentation Strategy for High-Resolution LA-ICP-TOFMS Bioimaging
Source: Anal Chem. 2025 Sep 18;97(38):20681–7. doi: 10.1021/acs.analchem.5c04060 (PMC12489891; doi:10.1021/acs.analchem.5c04060)
Supplement: Supplementary file 1 [file ac5c04060_si_001.pdf]

Supporting Information

Dual-Mode Wheat Germ Agglutinin Labeling – A Versatile Cell Segmentation Strategy for High-Resolution LA-ICP-TOFMS Bioimaging

Claude Molitor[1-3], Martin Schaier[1], David Loibnegger[1-3], Gabriel Braun[1,3], Michael Gutmann[4], Walter Berger[4], Gunda Koellensperger[1]*

[1] Institute of Analytical Chemistry, Faculty of Chemistry, University of Vienna, 1090 Vienna, Austria

[2] Institute of Inorganic Chemistry, Faculty of Chemistry, University of Vienna, 1090 Vienna, Austria

[3] Vienna Doctoral School in Chemistry (DoSChem), University of Vienna, 1090 Vienna, Austria

[4] Center for Cancer Research and Comprehensive Cancer Center, Medical University of Vienna, 1090 Vienna, Austria

* Corresponding authors:

Gunda Koellensperger

Institute of Analytical Chemistry, 1090 Vienna, Austria

Tel: +43-1-4277-52303, Email: gunda.koellensperger@univie.ac.at

Table of Contents

EXPERIMENTAL SETTINGS 3

FIGURES S1-S3 5

TABLE S1..8

REFERENCES 9

Experimental settings

Chemicals and Materials

Ultrapure water was prepared using an ELGA Purelab Ultra MK 2 system (High Wycombe, U.K.). Human BD Fc Block™ was obtained from BD Biosciences (San Jose, CA, USA). Dako Target Retrieval Solution (pH 9, Tris/EDTA) was purchased from Agilent Technologies (Waldbronn, Germany). SuperBlock™ blocking buffer (TBS) and Tween 20 were obtained from Thermo Fisher Scientific (Waltham, MA, USA). M-Xylene (anhydrous, ≥99%), Tris-buffered saline (BioUltra), ethanol (absolute, EMSURE®), and BSA were acquired from Sigma-Aldrich (Steinheim, Germany). The Ir-intercalator (Cell-ID™, 125 µM) was supplied by Standard biotools (San Francisco, CA, USA). Anti-Wheat Germ Agglutinin (Anti-WGA) from Szabo Scandic (Vienna, Austria) was labeled with the Maxpar® X8 Antibody Labeling Kit (^{143}Nd and ^{155}Gd) from Standard BioTools (San Francisco, CA, USA). The metal-labeled Anti-WGA's had concentrations ranging at 2 mg/ml. FITC-labeled WGA (conc. 5 mg/mL). DAPI staining solution (conc. 1 µg/ml) was purchased from GeneTex (Irvine, CA, USA).

Preparation of Human Skin tissue

The *ex vivo* skin model, NativeSkin®, was obtained from Genoskin (Toulouse, France). Biopsies originated from surplus adult skin collected post-surgery, with informed consent obtained from all donors prior to participation. The use of this model was approved by the appropriate Ethics Committee in accordance with relevant guidelines and regulations. Upon receipt, the supplied culture medium was applied under sterile conditions. The skin model was then incubated at 37°C with 5% CO_2 and 95% humidity for one hour, following the manufacturer´s instructions. After treatment, samples were collected, formalin-fixed, embedded in paraffin (FFPE). Tissue blocks were sectioned at a thickness of 5 µm.

HCT116 Cell Cultivation and Drug Treatment

The human colorectal cancer cell line HCT116 was obtained from the American Type Culture Collection (ATCC) and cultured in McCoy's 5A medium supplemented with 10% heat-inactivated fetal calf serum (FCS; BioWest). Cells were cultured at 37 °C in a humidified atmosphere containing 5% CO_2. For treatment, cells were seeded at a density of 0.5 x 10^6 cells/well in 6-well plates. After 24 h, cells were exposed to 25 µM oxaliplatin (OxPt; LC Labs; #O-7111) for 3h. Cell were detached using trypsin, centrifuged at 1400RPM, the supernatant removed, and cells were resuspended in 120 µL TBS.

LA-ICP-TOFMS Analysis

LA-ICP-TOFMS mapping was performed using an Iridia 193 nm LA system (Teledyne Photon Machines, Bozeman, MT, USA) coupled to an icpTOF 2R (TOFWERK AG, Thun, Switzerland). A low-dispersion cobalt ablation cell with an aerosol rapid introduction system (ARIS) optimized sample transport. The system was tuned on a daily basis with NIST SRM612 for high ion intensities, minimal oxide formation (<2.5% $^{238}U^{16}O^+/^{238}U^+$), and reduced elemental

fractionation. The instrumental parameters are summarized in Table S1. Spot sizes ranged from 1 to 2 µm circular spots with interspacing between 0.25 and 1 µm and a fixed dosage of 2 - 4 providing a 2x to 4x overlap in both the x and y directions to optimize ablation.

Data Acquisition and Processing

LA-ICP-TOFMS data were acquired using TofPilot (version 2.10.3.0, TOFWERK AG) and stored in hierarchical data format (HDF5). Data processing was performed with HDIP (version 1.8.5.171, Teledyne Photon Machines), using an automated script to generate two-dimensional elemental distribution maps. The resulting images were exported as TIFF files. Further data analysis, including cell segmentation, was conducted using the MeXpose image analysis pipeline, as previously described by Braun, Schaier et al.[1] To enhance visual clarity in figures, minimal image processing was applied in Fiji (v2.16.0/1.54p, NIH)[2], using Gaussian blur (σ = 0 to 0.6) and is stated accordingly.

Figures S1-S3

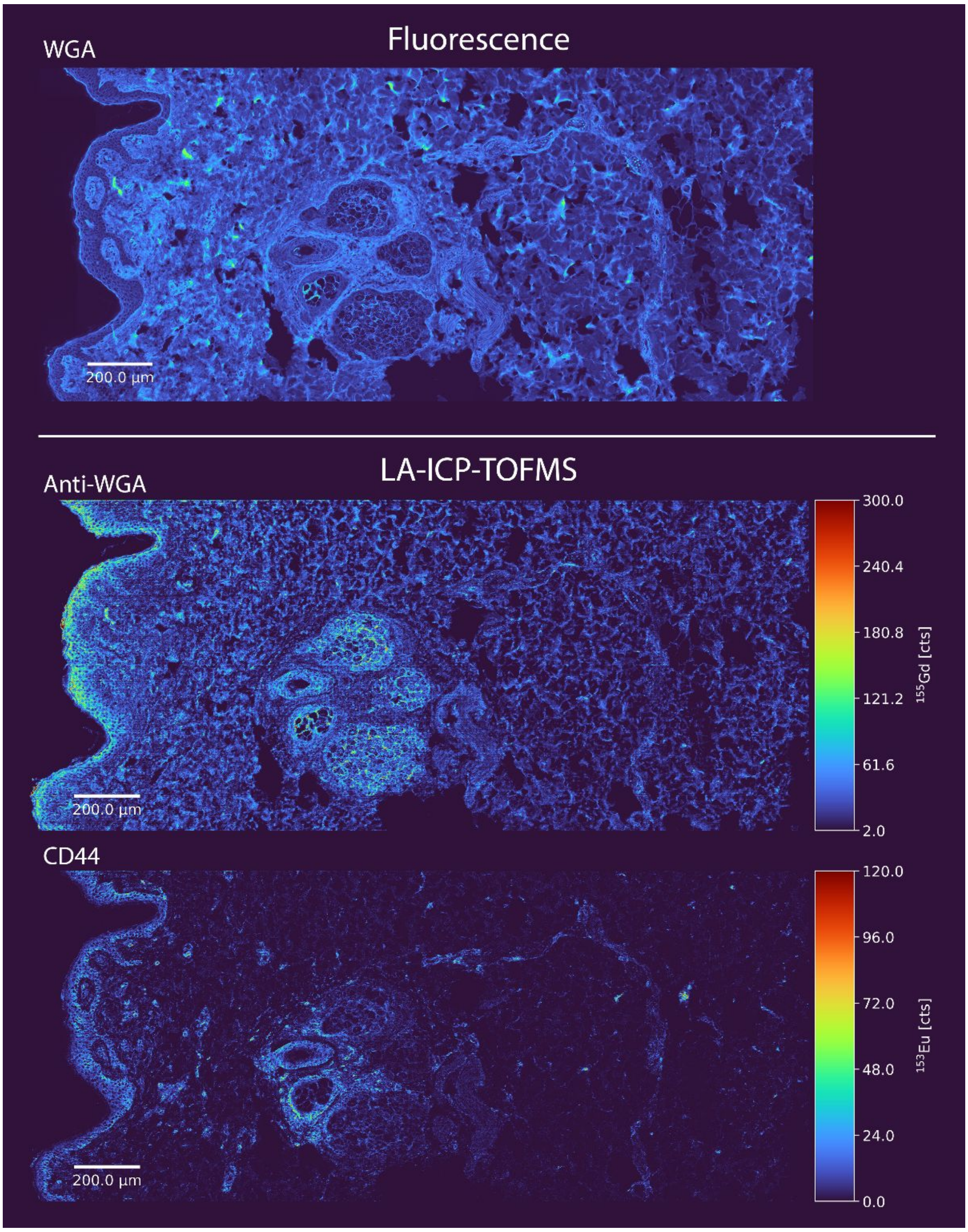

Figure S1: Large-scale comparison of IF and LA-ICP-TOFMS images of human skin samples after membrane staining using the F-WGA/M-Anti-WGA dual labeling and CD44 as established segmentation for skin marker on the same slide.

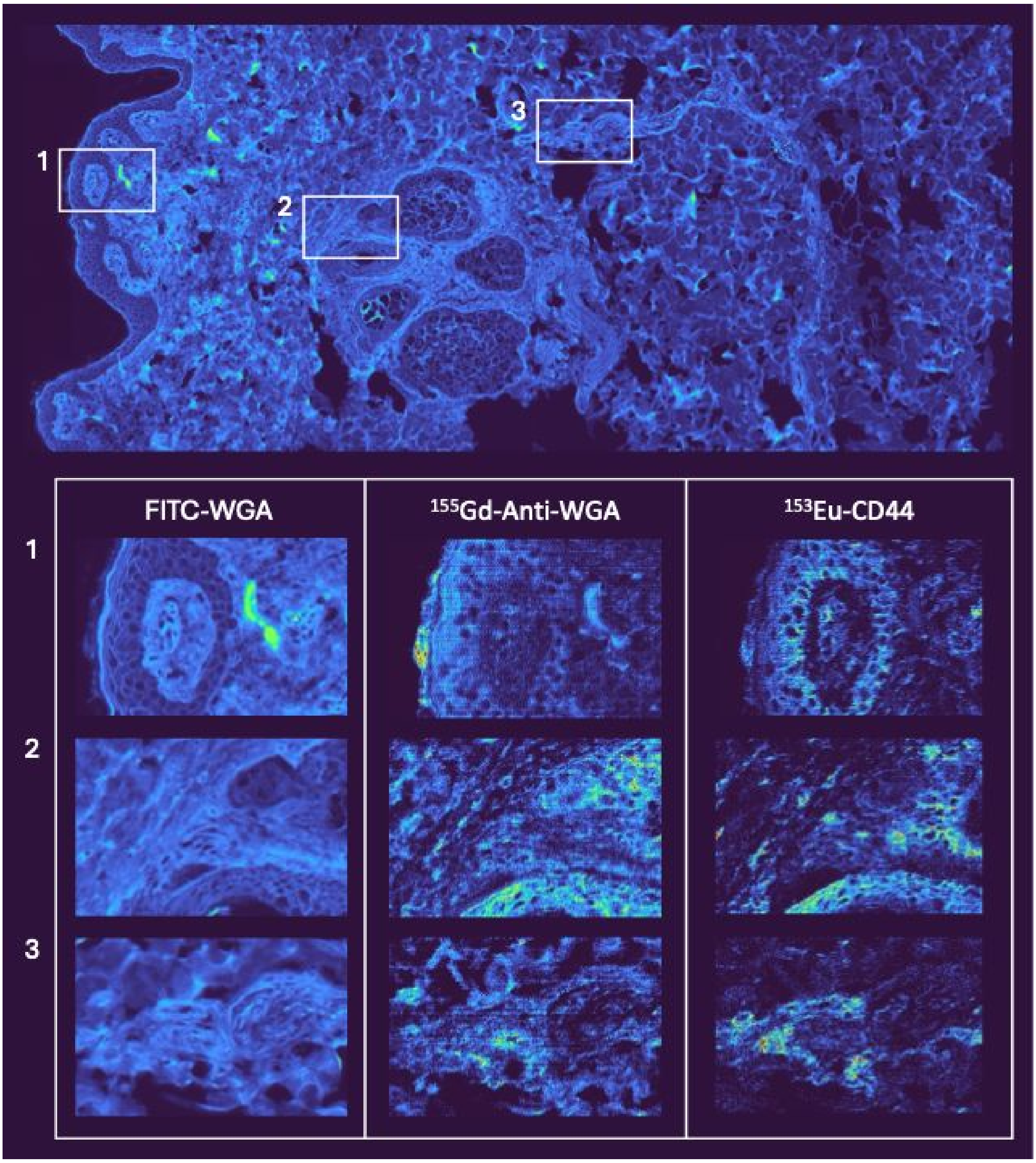

Figure S2: Close-up comparison of IF and LA-ICP-TOFMS images of human skin FFPE samples after membrane staining using the F-WGA/M-Anti-WGA dual labeling and CD44 as established segmentation for skin marker on the same slide.

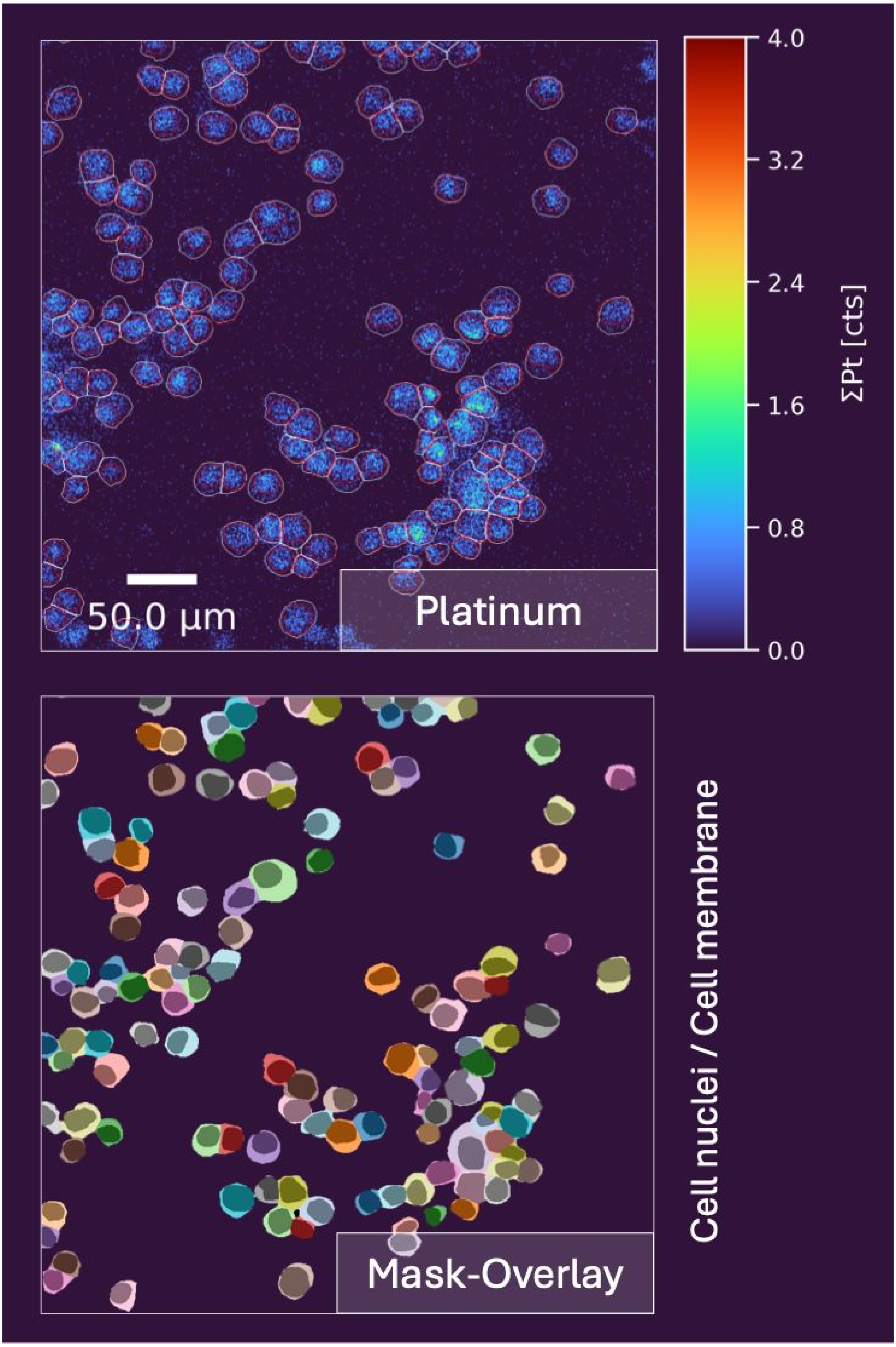

Figure S3: Close-up view of the platinum accumulation within the nuclei area compared to the cytoplasm as well as the segmentation masks overlay. White circles correspond to the cytoplasm mask; red circles correspond to the to the nuclei mask.

Table S1

Tabel S1: LA-ICP-TOFMS parameters

Parameter	
ICP-TOFMS	
RF Power [W]	1440
Sampling depth [mm]	3.5
Cone materials	Ni
Plasma gas flow [L min^{-1}]	14
Auxiliary gas flow [L min^{-1}]	0.80
Nebulizer gas flow [L min^{-1}]	1.06
Measurement mode	Collision cell technology (CCT)
CCT gas	93% He (*v*/*v*), 7% H2 (*v*/*v*)
CCT gas flow [mL min^{-1}]	4.2
m/z range	14-256
Laser ablation	
Spot size	1-2 µm (circular)
Interspacing (Y - Overlap)	0,25 - 1 µm
Repetition rate	250-500 Hz
Dosage (X - Overlap)	2-4
Shot count	1
Fluence	1.0-1.8 J cm^{-2}

References

(1) Braun, G.; Schaier, M.; Werner, P.; Theiner, S.; Zanghellini, J.; Wisgrill, L.; Fyhrquist, N.; Koellensperger, G. MeXpose–A Modular Imaging Pipeline for the Quantitative Assessment of Cellular Metal Bioaccumulation. *JACS Au* **2024**, *4* (6), 2197–2210. https://doi.org/10.1021/jacsau.4c00154.

(2) Rueden, C. T.; Schindelin, J.; Hiner, M. C.; DeZonia, B. E.; Walter, A. E.; Arena, E. T.; Eliceiri, K. W. ImageJ2: ImageJ for the next Generation of Scientific Image Data. *BMC Bioinformatics* **2017**, *18* (1), 529. https://doi.org/10.1186/s12859-017-1934-z.
